# Playing With Fire Compounds: The Tonal Accents of Compounds in (North) Norwegian Preschoolers’ Role-Play Register

**DOI:** 10.1177/00238309231161289

**Published:** 2023-04-27

**Authors:** Bror-Magnus S. Strand

**Affiliations:** UiT The Arctic University of Norway, Norway

**Keywords:** Phonology, child language, registers, phonetics, play

## Abstract

Prosodic features are some of the most salient features of dialect variation in Norway. It is therefore no wonder that the switch in prosodic systems is what is first recognized by caretakers and scholars when Norwegian children code-switch to something resembling the dialect of the capital (henceforth Urban East Norwegian, UEN) in role-play. With a focus on the system of lexical tonal accents, this paper investigates the spontaneous speech of North Norwegian children engaging in peer social role-play. By investigating F0 contours extracted from a corpus of spontaneous peer play, and comparing them with elicited baseline reference contours, this paper makes the case that children fail to apply the target tonal accent consistent with UEN in compounds in role-play, although the production of tonal accents otherwise seems to be phonetically target like UEN. Put in other words, they perform in accordance with UEN phonetics, but not UEN morpho-phonology.

## 1 Introduction and background

That children standardize their language in role-play is not uncommon (Buhofer & Burger, 1998; Kasperger & Kaiser, 2019; Katerbow, 2013; Sophocleous, 2013). This also holds for the Norwegian context, where children code-switch to something resembling the Oslo dialect (or Standard or Urban East Norwegian, UEN) in their “in character” role utterances (e.g. Røyneland, 2009, with the exception of children with that variety as their native dialect). This paper investigates tonal accents in the role-play register (RPR) of seven children (2;7–4;3) in the North Norwegian city of Tromsø, and, in particular, whether the children transfer the compound accent of their native dialect into their RPR or whether they master UEN’s system of differential accent marking in compounds.

Most varieties of Norwegian (and Swedish) have a prosodic system with tonal accents, referred to as accent 1 and accent 2. The distribution of the two tonal accents are similar across varieties when it comes to the interface between morphology and phonology and the marking of morphological material through tonal accents, with certain local differences (Kristoffersen, 2000). One difference is that in most Swedish and North Norwegian varieties, all compounds are assigned accent 2 by default, as opposed to most Norwegian varieties and some South Swedish varieties, where the first stem of the compound governs the tonal accent (Bye, 2004; Lorentz, 1981; Strandberg, 2014). In addition, the phonetic/acoustic features of the tonal accents, that is, their F0 contours, vary a great deal across varieties (Gårding, 1977). The phonetic differences between dialects are highly salient, and in line with the many reports of RPR in UEN (e.g., Bjørlykke, 1996; Høigård, 1999; Kleemann, 2015; cf. also discussion below), children could be expected to master the UEN tonal accent contours to some degree. This paper backs up these reports with examples of actual F0 contours from excerpts of children’s speech during spontaneous play. Building on that, the paper goes on to answer the question of whether the children have picked up on the distributional differences in compounds between UEN and their native dialect variety.

In the remainder of this section, an introduction to the Norwegian tonal accent system and the Norwegian RPR will be provided.

### 1.1 Tonal accents and prosody

In the Scandinavian tonal accent system, each lexical word is associated with one of two contrasting tonal contours (or pitch accents) (see, for example, Kristoffersen, 2000; Riad, 2014). This contrast is demonstrated by the existence of segmentally identical strings that are distinguished from each other by tonal accent only, as in *1’leken* (“the game,” tonal accent one, as indicated by the number preceding the stressed syllable) and *2’leken* (“the toy” or “playful,” tonal accent 2).^1^ The tonal accent contours are anchored to syllables carrying primary stress and span across unstressed material up until the next syllable, often splitting words or straddling word boundaries (Abrahamsen, 2003; Kristoffersen, 2000). Limiting the contrast to stressed syllables only, the tone distributions is much sparser compared with “canonical” tone languages, where every tone bearing unit (be it mora or syllable) can have a tone or a tonal contour (Gussenhoven, 2004). In many respects, the Scandinavian system is thus more similar to the pitch accent system of Japanese (e.g., Kubozono, 2008) than the lexical tone systems of Mandarin or Thai. Other typologically similar prosodic systems are found in Lithuanian (Kardelis, 2017), Latvian (Karins, 1996), Serbian and Croatian (Inkelas & Zec, 1988; Zsiga & Zec, 2013), as well as in varieties of Basque (Hualde, 2012), Korean (Cho et al., 2019; Jun et al., 2006; Lee & Zhang, 2014); Dutch (Ramachers et al., 2017) and Franconian German (Köhnlein, 2016).

**Figure 1.**
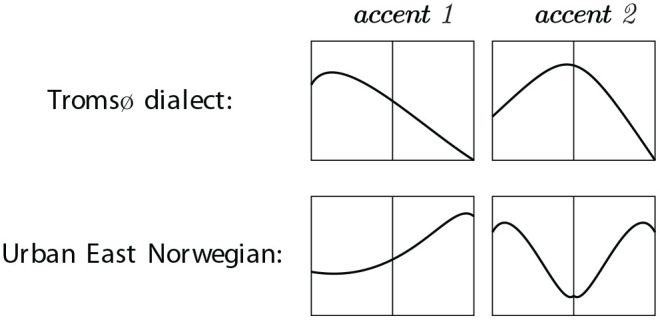
Schematic illustration of pitch contours of accent 1 and 2 in Tromsø dialect and Urban East Norwegian. The graphical representation is based on Bye (2004, p. 6), with reference to type “1A” and “2B” in the “Gårding-Lindblad typology” (Gårding & Lindblad, 1973). See also Almberg (2004) and Fintoft et al. (1978, p. 204) on the typology of Norwegian tonal accents.

**Figure 2.**
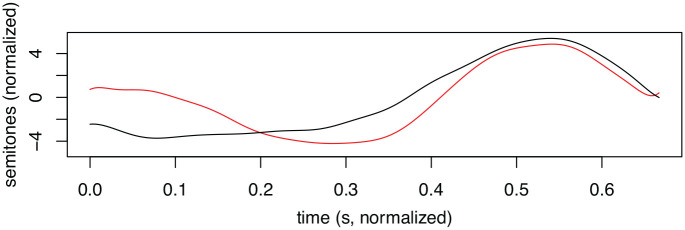
Example of F0 contours of two compounds, *2vann-mann* “aquarious” (red line) and *1brann-mann* “fire-fighter” (black line), in UEN, a d-dialect. The contours were smoothed and normalized according to the procedure outlined in Section 3.3.

The phonetic differences between Scandinavian dialects are often described in terms of the distribution of contour peaks (high tone, H) and valleys (low tone, L) across syllables and/or morae. The variation has been classified in various ways. Traditionally, varieties of Scandinavian have been grouped together based on whether the first stressed syllable in accent 1 words (also referred to as “acute”) is associated with a high tone (high tone dialects, for example, the Tromsø dialect) or a low tone (low tone dialects, for example, UEN). Another common division criterion is whether accent 2 words (also referred to as “grave”) have one or two peaks (Gårding, 1977; Gårding & Lindblad, 1973). Both criteria are relevant for the differences between UEN and the Tromsø dialect, which are schematically represented in Table 1 and illustrated in Figure 1. The distinction between accent 1 and 2 in the Tromsø dialect is mainly the placement of the H: in the first (stressed) syllable in accent 1, and across or on the syllable boundary in accent 2 (see Bye, 2004). In UEN, the distinction between accent 1 and 2 depends on the absence (accent 1) or presence (accent 2) of an H in the first syllable (the “lexical tone”), in addition to the H in the second syllable. (The F0 contours of UEN accent 1 and 2 are also given in Figure 2.) Note that the F0 contours of UEN and the Tromsø dialect are virtually opposites in terms of the distribution of Hs and Ls.

**Table 1.** Schematic Tonal Accent Contours in Urban East Norwegian (UEN) and the Tromsø Dialect in Bisyllabic Feet.

	Accent 1	Accent 2	Compound Accent
UEN	L* H	H* LH	
Tromsø dialect	H* L	LH* L	LH* L*

High (H) or low (L) tones marked with “*” are associated with the stressed syllable.

Another difference across Mainland Scandinavian varieties is whether all compounds are assigned accent 2 regardless of the individual constituents (henceforth “compound accent dialects,” or “c-dialects”), or whether the tonal accent of the first constituent determines the accent of the entire compound (henceforth “compound distinction dialects,” or “d-dialects”). Most Swedish and North Norwegian dialects are c-dialects, where compounds are assigned accent 2 by default (Bye, 2004; Lahiri et al., 2004; Lorentz, 1981; Myrberg & Riad, 2015). Therefore, the word pairs in (1)’Mahler-huset (“the house of Mahler”) and (2)’maler-huset (“the painter house”), and (1)’ball-rom (“ballroom”) and (2)’ball-rom (“ball pit”) have different accents in the d-dialects, as indicated by the parenthesized numbers, making the word pairs above minimal, whereas they all have accent 2 in the c-dialects, as do all compound words, making the word pairs above identical (disregarding any segmental dialectal differences). In the d-dialects, the accent of the compound is dependent on the first stem. For most compounds with polysyllabic words as the first stem, the mapping of pitch accent onto the whole compound in d-dialects is straightforward. This is, however, not the case for monosyllabic stems. Monosyllabic words have accent 1 in isolation, but as the *ball-rom* example above shows, this does not automatically map onto the whole compound. Precisely how the accent maps onto the compound word from monosyllabic first stems is opaque and does not always follow from the segmental properties or tonal accent of the roots. A thorough analysis of the system in UEN is given in Wetterlin and Lahiri (2012; see Kristoffersen, 2000 and Kaldhol & Köhnlein, 2021 for alternative accounts). The main stress is always on the first word in compounds in UEN and the Tromsø dialect.

There has traditionally been variation within UEN, as well as other dialects in Southeastern and Middle Norway, where Latinate and Greek loanwords, originally with (pen)ultimate stress, get initial stress, for instance *poli’ti* versus *’pol(i)ti* (“police”) (see Tengesdal & Lundquist, 2021). However, these variants have been in decline in UEN, and they do not seem to appear in the North Norwegian RPR. This is not a feature of the Tromsø dialect.

This paper has an empirical focus and the findings should be translatable into any theoretical framework for prosodic analysis. The formalism adopted for the annotation and description is the Trondheim model of prosodic analysis (Fretheim, 1981). Some important assumptions of this approach are the following: The domain of tonal accent is the Accent Phrase (AP), which encompasses the phonetic material from one stressed syllable (specifically, the onset of its nucleus) to, but not including, the next stressed syllable (the onset of its nucleus). As in other prosodic models (Nespor & Vogel, 1986/2012; Selkirk, 1984), the Trondheim model also supposes a prosodic hierarchy, where the AP is situated below the Intonational Phrase (IP) and above the prosodic word ( ω ).

The contours of the tonal accents vary in complex but predictable ways according to the position and function of the AP/tonal feet in the IP (e.g., Gårding & Stenberg, 1990). This makes comparisons between tonal accents or variants difficult unless a number of other prosodic variables are controlled for. For instance, as has been described thoroughly in Fretheim (1987), low tone feet can appear similar to high tone feet in compressed declines, as often found post focally. Since this investigation depends on precisely the difference between high tone and low tone F0 contours, compressed APs are uninformative to this analysis, and will not be included in the analysis.

#### 1.1.1 Acquisition of lexical tone and tonal accents

Most studies of child acquisition of tonal languages have been done on Mandarin, Cantonese, and Thai (and, in some cases, Yoruba) (see Singh & Fu, 2016, for a review). These tone systems are of course very different from the tonal accent (or pitch accent) system of Scandinavian, both in terms of number of tones (4, 5, and 6, for Mandarin, Thai, and Cantonese, respectively) and in how they interface with the overall prosodic system (as laid out above). This means that the results from these studies cannot automatically be taken to hold also for the acquisition of Scandinavian tonal accents. However, it is of theoretical and practical value to discuss them here, nevertheless.

Compared with the ability to discriminate between non-native vowels and consonants, the time window during which children are sensitive to and can discriminate between lexical tones in other languages than their native one seems to close earlier (Singh & Fu, 2016). However, it is also reported to open again, thus indicating a U-shape: The ability of learners of a non-tone language to discriminate tones has been reported to decline around 8 months, and then increase again around 12 months, which stands in contrast to vowel and consonant discrimination, which is reported to decline at 10–12 months (Singh & Fu, 2016). For native acquisition, children seem to “show primitive lexical tone categories . . . as early as 4 months” (Singh & Fu, 2016, p. 840), in general earlier than the acquisition of vowel and consonant categories. A possible explanation for this can be the “periodicity bias.” That is, that infants are particularly attuned to voiced sounds. Indeed, suprasegmental features are the only area of grammar where acquisition is demonstrated to happen even prenatally (e.g., Moon et al., 2013). The window seems to close again for non-tone language learners after 19–24 months, where informants will accept “tone variants as differing realizations of the same word” (Singh & Fu, 2016, p. 841). Conversely, bilingual tone language learners are able to disregard tone when parsing words in their non-tone language from around 11 to 12 months (Singh et al., 2016; Singh & Fu, 2016). The salience of the contrast between two tones seems to determine how well and how early young tone language learners are able to use them in word detection (Singh & Fu, 2016).

As for production, in studies where adult raters have been used, children have been reported to display very few tone errors after 1;6 or 2 years, for Mandarin and Cantonese, respectively. In contrast, when digital speech analysis and manipulations are used, researchers have found that even at 3 years of age, Mandarin and Cantonese learning children perform both quantitatively and qualitatively different from adults (Wong, 2013; Wong et al., 2005). Keep in mind that these studies only analyzed F0, and not any of the secondary cues to tone (Singh & Fu, 2016), and that these systems are quantitatively and qualitatively different from Scandinavian.

A system that is much more similar to the Scandinavian tonal accent is the Japanese system of pitch accents. Based on a study with a preferential looking design investigating 17-month-old infants learning Tokyo Japanese, Ota et al. (2018) argue that children at 17 months “are still in a nascent state when it comes to” (p. 10) pitch accents, although children are found to be able to discriminate the same contrast at 4 months (Sato et al., 2010). They further argue that this delay is due to very variable realization of the pitch accent across different contexts. In a novel word learning task with Dutch toddlers (2;5–4;0), Ramachers et al. (2017) found that toddlers acquiring a Dutch dialect *with* tonal accent (Limburgian) and *without* tonal accents (Standard Dutch) paid attention to the accent cues in the novel word learning task, as assessed through eye-tracking.

Finally, we can also review the relevant Scandinavian studies: command of the tonal accents in speakers’ native variety is reported from a very early age. Romøren (2011) reports that Norwegian children between 29 and 36 months produce the correct tonal accent around 90% of the time. Kadin and Engström (2007, p. 70) report that Swedish 2-year-olds produce the accent contrast.

#### 1.1.2 Imitation and metalinguistic awareness

Since the current study investigates the adaptation of features of a capital, arguably “standard,” variety (see, for example, Røyneland, 2009), it bears resemblance to socio-linguistic and variationist concepts such as accommodation and convergence. Although the RPR will be discussed below, an assessment of the relevant parameters of prosody and tone in accommodation and imitation can aid us in the discussion of the results.

The terms accommodation, shadowing, and imitation overlap in the literature, and all refer to ways in which speakers adapt their speech as an effect of spoken stimuli, either from an interlocutor or experimental stimuli. In phonetic accommodation and imitation, one of the variables that seems to influence whether a feature is imitated is the distance between the dialect of the imitator and the model speaker: the longer the distance (up until a certain point), the more imitation (Lin et al., 2021). D’Imperio and German (2015) find an effect of exposure to the shadowed variety in prosodic imitation of American English by Singapore English speakers. There is also a discussion to be had of whether imitation is constrained by the phonological contrasts in the speakers’ first language, but the results here are mixed (Braun et al., 2006; D’Imperio et al., 2014, 2015; German, 2012). Interestingly, metalinguistic awareness of the feature in question does not seem to be a necessary condition for imitation to happen (Lin et al., 2021). Furthermore, Petrone et al. (2021) found that “speakers with higher working memory capacities were more accurate in phonological imitation” (p. 1), whereas no such effect was found for phonetic imitation. Somewhat similarly, Bosma et al. (2017) found a correlation between scores on verbal working memory tasks and the acquisition of cross-linguistic linguistic regularities across two closely related languages in Frisian–Dutch bilingual children (5–8 years old).

To the author’s knowledge, Lin et al. (2021) is the only example to date of a study of imitation of lexical tones. In their shadowing study in Hong Kong Cantonese, they found that shadowers with an ongoing but not complete merger between two tones reversed the merger when shadowing a speaker with a clear distinction between the tones in question.

Although no-one seems to have investigated imitation or shadowing of tonal accents or other prosodic or suprasegmental variables in Norwegian, a study that bears some resemblance is van Ommeren and Kveen (2019): with data from a corpus of sociolinguistic interviews of Norwegian bilectals, they investigate speakers’ examples and meta-linguistic descriptions of the supra-segmental phonological features of their different dialects to identify the lay or folk-linguistic concept of *tonefall*, (which loosely translates into “intonation”) and which part(s) of supra-segmental phonology the term seems to refer to. They argue that the informants have the ability to exemplify and pinpoint prosodic features and know the geographic distribution of these. Indeed, prosody is often considered as the most salient dialect feature when distinguishing between Norwegian dialects (Kerswill, 1994; Mæhlum & Røyneland, 2012; van Ommeren and Kveen, 2019).^2^ For instance, in an experimental perception study, Fintoft (1970) finds that citizens from five Norwegian locations^3^ are able to identify the correct tonal accent (accent 1 as 1 and 2 as 2) in each others’ dialects (and their own) to degrees between 83.9% and 97%, and that the tonal accents in the “Oslo dialect” (i.e., UEN) were always correctly identified by all groups. This indicates that Norwegians have a good perceptual knowledge of the tonal accents in UEN. Despite its alleged salience, Van Ommeren and Kveen (2019) point out that very little interest has been devoted to the study of Norwegian prosody in a variationist or sociolinguistic perspective, at least compared with the number of studies looking at the variation in segmental and morphological features.

To sum up this section, tonal accents and other prosodic features are highly salient features of Norwegian language variation, which speakers seem to have a meta-linguistic awareness of. However, looking at studies in imitation and accommodation, meta-linguistic awareness does not seem to be an important factor, as opposed to exposure and linguistic distance, as well as the imitators’ working memory capacities. That being said, keep in mind that the RPR is not direct imitation in the strict sense: children are not accommodating or imitating anyone or anything present in the role-play.

### 1.2 RPRs

There are several reports that children standardize their language or code-switch to a standard variety during in character role utterances. The phenomenon is reported in the literature from Germany (Katerbow, 2013, on Franconian dialects), Austrian (Kasperger & Kaiser, 2019, p. 333), Switzerland (Buhofer & Burger, 1998), Scania in Sweden (Lindström, 2002) and among Swedish speaking children in Ostrobothnia, Finland (Östman & Mattfolk, 2011, pp. 89–90), and in Cyprus (Sophocleous, 2013). In addition, the present author has encountered anecdotal reports of similar phenomena from both Japan and rural Argentina. Several of the relevant dialect areas do also have tonal or pitch accent systems, at least Swedish, Japanese, and Franconian dialects in Germany. Thus, the methodology and findings from this study should have direct relevance for other situations. Norwegian children have been known to code-switch to something resembling Urban East Norwegian (Allern, 1995, Åm, 1989; Bugge et al., 2017; Røyneland, 2009; Venås, 1983). We know that the RPR involves all or most domains of language, but there are many aspects of this variety that still need to be explored. The reason why children code-switch to a different dialect is subject to speculation. One recurring hypothesis is the need to signal “otherhood” (Bjørlykke, 1996; Høigård, 1999; cf. *metacommunication* in, for example, Bateson, 1955/1976).

Although Norwegian children’s RPR is widely and colloquially recognized as UEN in Norway, also in popular literature, there are few studies looking into its structural properties (but see Eliassen, 1998; Strand, 2020a). The register seems to be acquired with little to no input of the kind often argued to be necessary for natural first-language acquisition to take place. The presence of a speaker of the standard variety in the immediate daily environment is no prerequisite. As there is no codified spoken standard in Norway (Vikør, 1993), no formal instruction is given either, and the onset of the UEN-like RPR precedes literacy training in school (Strand, 2020a). Television and caretakers’ reading of books have been suggested as possible input sources. However, in addition to being scarce, non-ideal input sources of language, TV cannot explain the anecdotal and literary reports of UEN as an RPR that pre-dates broadcasting (e.g., Høigård, 1999), and reading of books to children cannot explain the reported use of UEN prosody. Children’s main source of input for this register seems to be peers in play groups, although the original source of the linguistic material must be something else (see Strand, 2022, for a longer discussion).

Most descriptions or reports of Norwegian children’s RPR do not mention specific domains of the language system, but often limit themselves to reporting that children code-switch to Urban East Norwegian (or an equivalent term). If prosody is mentioned at all, it is in passing (Kleemann, 2015) without specifying the extent to which this behavior is target-like or which part(s) of the prosodic hierarchy it holds for, or backing this up with linguistic data. As their focus of investigation has lain elsewhere, this is no wonder. An exception is Eliassen (1998), who includes relevant examples in her qualitative study of the (North) Norwegian RPR. Eliassen (1998, p. 143) notes that North Norwegian children tend to use “low tone”—the contour of UEN, among other varieties—(see Section 1.1) in their role-play utterances, but that some utterances can start in East Norwegian intonation and end in Northern Norwegian intonation. In the case study of one of the recordings, she illustrates these observations with F0 contours (pp. 93–97).

Although the consistency and the degree to which children code-switch in role-play seems to vary (Strand, 2020a), the children have a certain bilectal competence. The nature of this bilectal competence is of theoretical relevance, as it informs us of children’s ability to acquire a second-language variety with non-typical language input. Furthermore, it can potentially inform us about how closely related varieties are organized in the mind. UEN is in many regards a spoken equivalent of the majority written standard. Thus, the investigation of children’s competence in UEN prior to any literacy training has a practical relevance.

To the author’s knowledge, the only study looking at the structural linguistic competence in children’s RPR and linking it to language acquisition in this way, is Strand (2020a), which looks at verb morphology and pronouns, based on the same corpus as this study. Strand (2020a) reports variability in the use of UEN (referred to as “Standard East Norwegian” in Strand, 2020a), but with an increase toward more UEN variants as an effect of age. Furthermore, Strand (2020a) reports that some variables are used more consistently in the UEN variant than others. Salience is argued to be a possible reason for this, either conceptual (semiotics) or statistical (frequency) or a combination of the two.

Adult Norwegian speakers have a high metalinguistic awareness of prosodic features, perhaps unmatched by most other linguistic features. It is therefore no wonder that caretakers and scholars pick up on the prosodic features of the RPR more readily than other features.

If children use the RPR to signal “otherhood,” their code-switching from their local dialect variety to something different can be seen, in variationist terms, as the children diverging from their normal self, or *auto-divergence*. This is a good fit with the postulates of “third wave” sociolinguistics (Eckert, 2018), where speakers are viewed as using semiotic resources, such as language, actively in identity-construction and -maintenance, in taking on and embodying different personae. Since salience has proven to have some explanatory power in studies of language convergence and divergence (Kerswill, 1994; Trudgill, 1986), one should expect prosody, including tonal accents, to be one of the features first adopted by children when auto-diverging in role-play, although it has hitherto not yet been tested empirically or experimentally.

## 2 Aim of the study

From the reports of UEN prosody in RPR of Norwegian children, we can conjecture the following underlying Hypothesis A.

- Hypothesis A: The UEN prosody in children’s RPR is “target-like.”

The suprasegmental features that can broadly be defined as encompassed in the term “prosody” have (a) acoustic features and (b) variation governed by the interface with other parts of the language system (morphology, syntax, pragmatics, etc.), which all in theory can be measured by the extent to which they match the UEN target. In this study, Hypothesis A is put to the test at the interface between morphology and prosody, and specifically the variation between compounds and simplex words that distinguish the c-dialects from the d-dialects. This is summed up in the following research question:

- Research question: To what extent is the morpho-phonological distribution in children’s RPR “target like” Urban East Norwegian?

This can be investigated by comparing the F0 contours of APs with compounds in the children’s spontaneous production in peer role-play to that of a relevant baseline reference.

For this investigation to be valid, the children’s production should be investigated to ascertain that they use (both of the) UEN pitch accent contours in their RPR. In other words, the reports of children’s use of UEN prosody should be put to the test by inspecting their produced F0 contours in role-play. Based on the literature reviewed above, we would expect them to have a certain command of the phonetic/acoustic features of the UEN dialect. Given the degree to which Norwegian speakers have (implicit) perceptive knowledge of the UEN variety (Fintoft, 1970), and the fact that they seem to have metalinguistic awareness of some of its relevant features (van Ommeren & Kveen, 2019), it would be remarkable if the RPR prosody, which has been reported to sound like UEN, turned out to be nothing like it upon closer inspection.

Regarding the morpho-phonological distribution, it is not as easy to try to predict the degree to which children’s production will be target like. On the one hand, children do have an early command of the tonal accent in their native dialect (although dialect differences may play a role here). On the other hand, it is not clear how much exposure the children have to UEN, especially from native speakers, and there is a question of whether the children will have had the necessary input available to pick up the differential marking in UEN. An additional question is whether UEN is the “target” for the RPR (in its entirety). The RPR, as a metacommunicative code used to auto-diverge in role-play, may be a collection of features sufficiently emblematic and salient to signal “otherhood” which includes the phonetic/acoustic features, but not the morpho-phonological distribution. This opens up for the possibility that children “know” the system and have the ability to process and produce the tonal accents correctly, but do not produce it in role-play because it is not a part of that code. As this study only looks at production in RPR, it cannot inform us about this question, but Section 6 points to some possible roads ahead.

Since RPR is inherently creative and variable, quantifying the degree to which the average of APs in the corpus compare with the “target” UEN tonal accents would not be very informative, and it is not straightforward how this could be measured. Children’s competence in play is dependent on the specific role, play setting, and utterance type. Even within groups of utterances that can be coded as “in-character role play,” an overall average of the APs would not inform us of their competence in RPR or UEN. In this study, the question is not whether the children perform target-like UEN in all APs (or the extent to which they do so), but rather if those APs that are perceived as UEN upon closer inspection sound (or look, according to F0 contours) target-like, that is, certifying that the reports hold up. After that is established, the compound APs can be investigated. Only the compound APs that are perceptively uttered in UEN prosody are investigated, since there is no point in comparing compound APs that are uttered in the native North Norwegian dialect prosody, albeit in a role-play setting: the APs uttered in children’s native (c-)dialect are not expected to show differential compound marking, and it would not make any sense to investigate their correlation with the UEN baseline reference APs. In summary, it would not inform us about the research question. For practical reasons of analysis that will be elaborated in the following section, only compounds starting with *brann* (“fire”) will be subject to analysis.

## 3 Participants and method

In this study, excerpts from a corpus of spontaneous role-play among preschool North Norwegian children are investigated and compared with elicited speech from adult UEN speakers. In this section, the corpus data and the transcription, coding, and analysis procedure are presented.

### 3.1 Corpus data

The data are obtained from a corpus of recordings of free play and interaction between seven typically developing children. The recordings are conducted over a year in a kindergarten in Tromsø, North Norway. The data on compound accents are excerpted from the whole corpus (ages 2;7–4;3, see Table A1 in Appendix A), whereas the data on APs with simplex words are gathered from a smaller subset of the corpus where the children were around 42 months old (see Table 2), so as to limit the amount of data.

**Table 2.** Files and Ages for Simplex Data.

Subject:	Celice	Lars–Lars	Inga	Morten	Hedda	Kimbo	Klara
File No.	16	16	18	9	16	21	25
Months	42	42	43	42	44	43	42
Utterances	199	252	316	364	381	112	208
Role utterances	139	168	73	200	255	34	66

The data were transcribed by the author and/or a research assistant in ELAN (Brugman & Russel, 2004; Sloetjes & Wittenburg, 2008) using a semi-phonetic transcription system developed for a group of corpora in the LIA project (Hagen et al., 2018; LIA, n.d.). The transcriptions were then coded for level of pretense (i.e., “role utterance,” “planning utterance,” “everyday utterance,” etc.). For an utterance to be labeled as role utterance, one of the following criteria had to apply (see also Strand, 2020a):

1. The utterance is clearly referring to something not happening in the “baseline” reality, (e.g., “I am peeing” or “the . . . is on fire!”), and/or
2. The utterance is uttered with a voice quality or intonation that was clearly manipulated in a creative way so as to indicate role utterances, and/or
3. The utterance is uttered while holding or animating a doll, and/or
4. The utterance is uttered as an answer to or in a conversation together with an utterance with the characteristics in 1–3.

In unclear cases, the utterances were coded as uncertain. The coding procedure involves a certain amount of interpretation on the part of the coder, which is a possible source of error. To amend this, the anonymized transcription files are available in Strand (2020b)—the replication data for Strand (2020a)—and anonymized excerpted sound files, with transcriptions, are available in Strand (2021), the replication data for the present paper.

As there was a toy fire tank engine, toy firefighters and a toy fire station present in most of the recording sessions, compounds with “fire-” (*brann-*) are relatively frequent in the corpus. The nouns *brann* (“fire”) and *vann* (“water”) have identical rhymes and both have a default accent 1, as do all monosyllabic words in both dialects. Compounds with these nouns as first components, however, differ in accent in d-dialects: compounds with fire- (henceforth “fire- compounds”) have accent 1, whereas compounds with water- (henceforth “water- compounds”) have accent 2. In c-dialects, they both have accent 2. This makes the fire- compounds as ideal testing grounds for the present RQ, as they can easily be compared with both fire- and water- compounds as uttered by native speakers of UEN, due to their shared segmental properties.^4^

The language in role-play is highly creative and varied, as it is used both indexically, to mark the identity, stance, and sentiment of role characters (Andersen, 1990/2014; Auwärter, 1986; Sachs & Devin, 1976), with illocutionary force, to call items, characters, actions, and events into existence when setting the narrative (Kaper, 1980; Lodge, 1979; Strömqvist, 1984), and metacommunicatively, in conveying for which reality (“baseline” or “pretense”) the truth value of the utterance is meant to hold (Bateson, 1955/1976). This creativity in language influences the prosody, for instance, in agitated shouting in panic (e.g., from a doll house ablaze) and in sing-songy utterances (“ludic speech acts,” see Strömqvist, 1981). Utterances with such characteristics do not inform the research question and are left out of the analysis.

### 3.2 Reference baseline data

As a reference baseline for the comparison of F0 contours of the children’s RPR to UEN, utterances with fire- and water- compounds were elicited from five participants (two women and three men) from in or around the Oslo area, who all agreed that their native dialect variety could be characterized as UEN. These speakers all had some or extensive training in linguistics. The recordings were made using the participants’ own cell phones and coded and analyzed in the same manner as the children’s utterances.

The accent phrases were extracted from sentences of the type “No, it was a FIREMAN, that I saw in the firetruck, I said!”/”No, it was a WATERMAN, that I saw in the watertruck, I said!” (see Table B1 in Appendix B) alternating between contrastive (focal) and no focus (non-focal). Six pairs of different inflections of three fire- and water- compound words were excerpted, in order to cover the properties of as many APs from the corpus as possible, regarding number of syllables, stress patterns, and distribution of long and short vowels. The different word forms are given in Table 3. This sums up to a total of 24 categories (6 word forms * 2 ( fire- and water-) * 2 (focal, non-focal)).

**Table 3.** Different Word Forms in the Reference Baseline Data.

Compound	Meaning	Phonological properties
*brann*-/*vann-mann*	“fireman”/“water-man, Aquarius”	′V′V
*brann*-/*vann-mannen*	“the fireman”/“the water-man, Aquarius”	′V′VV
*brann*-/*vann-mennene*	“the firemen”/“the water-men”	′V′VVV
*brann*-/*vann-bil*	“firetruck”/”water-truck”	′V′V:
*brann*-/*vann-bilen*	“the firetruck”/“the water-truck”	′V′V:V
*brann*-/*vann-stasjonen*	“the fire-station”/“the water-station”	′VV′V:V

“V” and “V:” indicate syllables with short and long vowels, respectively, “ ′ ” indicate original stress position in the roots.

In the utterance of final vocative expressions, the noun (phrase) tends to lose its primary stress. As a result, it gets embedded in an AP together with the preceding primary stress, becoming a part of a new, larger accent contour instead of getting its own (compare *du er* (2)[*dårlig, brannmann*]AP (“you’re bad, fireman”) vs. *du er en* (2)[*dårlig*]AP (1)[*brannmann*]AP (“you’re a bad fireman”) see Fretheim, 1988, p. 64). This means that the utterance of final vocative fire- compounds will not inform our research question and are left out of the analysis.

### 3.3 Coding and analysis

Both the excerpts from the corpus and the reference baseline data were analyzed in Praat (Boersma & Weenink, 2020). Based on a trial and error approach, the voicing threshold was set somewhat lower than the standard setup (0.35 rather than 0.45), as it gave more reliable data and reduced the need for manual corrections (more on this below). The sampling rate was set to 0.01 second (i.e., 100 measure points/second). The F0 contours were subject to minor manual corrections (correcting errors made by the algorithm, where it had chosen an incorrect harmonic, or devoicing where unvoiced segments were analyzed as voiced, e.g. due to the reverb of the room or mechanical noise with periodic features). The syllable boundaries of the accent phrases were coded based on visual and aural inspection in Praat, and based on the theoretical framework in Kristoffersen (2000) (onset maximization and geminates divided across syllable boundaries).^5^ The accent phrases were analyzed for prosodic features (e.g., terminal contours, decline, focality).

For the fire- compound APs from the corpus to qualify for analysis, they had to meet the following criteria: (a) they had to be in UEN, that is, not their native dialect or agitated screaming, coded by the researcher),^6^ (b) their F0 data had to be salvageable (i.e., modal voice and not too much background noise or other children speaking at the same time), (c) they could not be in a compressed prosodic decline or (d) in an utterance final vocative (see above), and (e) they had to have the same number of syllables as one of the six baseline reference APs. Terminal contours (high or low) were not taken into account, assuming discrepancies in relation to the baseline data would be equally penalized in comparison to both fire- and water- compound contours and have no effect on the result. Criteria (b) and (c) also hold for the baseline reference data.

**Figure 3.**
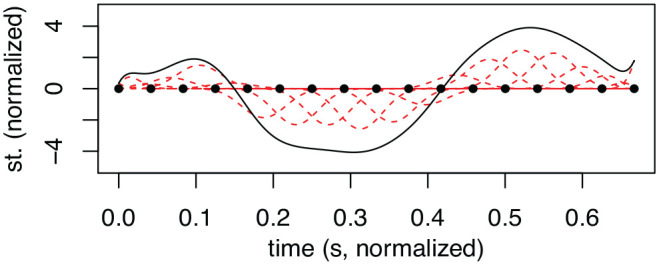
Example of F0 contour (black line) with B-spline basis (red lines), and B-spline knots (black dots), given in semitones (st.).

To make the quantitative analysis of F0 values of tonal accent contours valid, the contours have to be normalized so that the onset, offset, and syllable boundaries of the individual AP are aligned to amend for differences in speech rate and segmental features in the AP not relevant for the investigation. In this study, a set of tools from a class of statistical analysis known as *functional data analysis* (FDA, Ramsay & Silverman, 2005) have been used to this end. As some readers may be unfamiliar with this class of statistical analyses, it warrants a short introduction here. Values (e.g., F0) distributed along some dimension (e.g., time) can be analyzed as (wave or bi- or poly-nomial) functions. These functions, in turn, can be subjected to statistical analyses that better take into account the functional aspects of the signal. By analyzing F0 contours, such as tonal accents, in terms of functions instead of (post)theoretic constructs such as the timing of a peak or value in relation to some independent time domain (start/end of foot or syllable), differences and similarities between the accent contours can be analyzed in a pre-theoretic manner. For the current FDA analysis, basis-splines (B-splines) were used. B-splines can be characterized as an array of polynomials, each spanning overlapping points (knots), where the sum of the polynomials approximates the signal. The degree of fit to the signal depends on the degree (quadratic, cubic, etc.) and density of the polynomial bases, as well as a linear differential operator controlling the flexibility of the curves. In Figure 3, the basis (red) and knots (dots) as well as the smoothed signal (black) of a tonal accent contour are given.

Gubian and colleagues (Gubian, n.d.; Gubian et al., 2015) have pioneered the use of functional data analysis on acoustic measures in phonetic research. Rather than explorative statistical procedures applied in the cited papers (e.g., Principal Components analysis), a functional correlation coefficient (CC) equivalent to Pearson’s product–moment correlation coefficient (Li & Chow, 2005) is used to compare the children’s accent phrases with those in the baseline reference data.^7^ The CC ranges from −1.0 to 1.0, 1.0 being a perfect correlation. There is a certain tradition for using correlation coefficients in research on prosody, for example in reading prosody research (Benjamin & Schwanenflugel, 2010; Miller & Schwanenflugel, 2008; Schwanenflugel et al., 2004). The rationale for using correlation coefficients in the present study is that if the child uses a target-like tonal accent, the accent’s CC with the target baseline contour (fire- compound, accent 1) should be higher than the CC with the non-target baseline contour (water- compound, accent 2), and vice versa if the child overgeneralizes the contour tonal accent (≈ accent 2).

In this study, smoothing and timing algorithms from functional data analysis were used, specifically from the package fda (Ramsay et al., 2009, 2020) in R (R Core Team, 2019), building on the procedure in Gubian et al. (2015). The Praat and R scripts are available in the replication data repository (Strand, 2021).

Although FDA in principle is capable of handling data that are unevenly sampled, the placement of knots of the B-spline basis (start and end of each spline, see Figure 3) is fragile to missing data points. To retain comparability across APs with and without unvoiced segments, the B-spline knots were placed evenly across syllables rather than outside of unvoiced sections. To amend for the missing data points at the knots at unvoiced segments, the F0 signal was interpolated (linear) across unvoiced segments, done automatically in the R script.

No statistical inferences are made in this paper, and only descriptive statistics are reported for the following reasons: it is difficult to establish a confidence interval (CI), i.e., the threshold beyond which the null hypothesis (e.g., “the children’s compound F0 contours are (not) target like UEN”) would be considered falsified. It is not sufficient to test the statistical correlation (or lack thereof) of two sets of data points, each representing an F0 contour. A scientifically meaningful comparison can only be made in terms of the connection to linguistic structure, that is, accent 1 and 2, and speakers’ recognition of them. This is not a straightforward connection to make. In addition, the paper is primarily exploratory, which is known to inflate the possibility of false positives (type I errors) in null hypothesis significance testing (Roettger, 2021).

## 4 Results

In this section we illustrate the use of UEN-like tonal accents in the children’s RPR, as has frequently been reported in the literature (see Section 1.2), before we review the baseline reference data, and give the analysis of the compound data from the corpus. Keep in mind that the inspection of the simplex (non-compound) words and investigation of the compounds regard different linguistic levels. For the simplex words, we inspect whether children’s tonal accents in their RPR *phonetically* resemble UEN tonal accents. Here, we place confidence in the literature, and examples of F0 contours of APs from the child informants are deemed sufficient to illustrate the fact that children have some command over a register with tonal accent contours that differ from their native dialect and share properties with UEN. This prepares the ground for the subsequent analysis of the *morpho-phonological* properties of the fire- compounds. Being a specific subset of the utterances makes the compounds eo ipso a more controlled setting amenable to quantitative analysis and discussion.^8^

### 4.1 Simplex words

**Figure 4.**
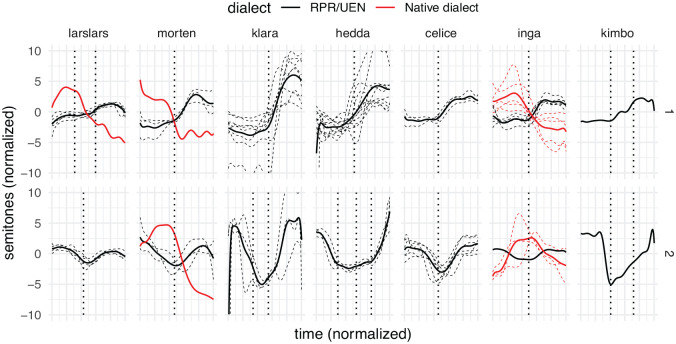
Examples of contours in RPR (black lines), with syllable boundaries (dotted vertical lines), accent 1 in top row, and accent 2 in bottom row. The mean F0 for each AP group is given (solid lines) alongside individual contours (dashed lines). To illustrate the clear difference between the UEN and Tromsø dialect tonal accents and thus the degree of code-switch between the two, examples of contours from the children’s out-of-play utterances have also been included for some of the children (red lines).

The values of the contours were normalized (i.e., the mean pitch in semitones has been subtracted to center the contours around 0). The contours were smoothed and their timing was normalized according to the procedure outlined in Section 3.3, so that the total duration and placement of syllables have been synchronized within groups (based on subject, accent, and number of syllables). In Figure 4, examples of F0 contours from the seven children are given. The figure presents all UEN contours for each speaker and accent with a certain number of syllables, so it is not exhaustive. For some of the children, examples of APs in their native Tromsø dialect are given as well for comparison. The total durations have been synchronized across groups for exposition.

The acoustic manifestation of the tonal accent contours varies between Scandinavian varieties: there is a difference in whether the second/last syllable is a peak (for instance UEN, see 2) or a valley (for instance, the Tromsø dialect), and whether accent 2 has two peaks (for instance, UEN) or one peak (for instance, the Tromsø dialect). Even to linguistically untrained Norwegian ears, most of the role utterances will be recognized as something resembling UEN, and not as Tromsø dialect. The contours presented in Figure 4 show features of UEN tonal accent prosody, with a single peak in the last syllable in accent 1 (first row), and two peaks, in the first and last syllables, in accent 2 (second row).

The examples of F0 contours given in Figure 4 do not serve justice to the variation and creativity in the children’s production. Although the main impression is that the children use UEN prosodic features, there are examples of switching in prosodic systems within role-play. The switching includes transitions within utterances (as reported in Eliassen, 1998), and code-switching that can be interpreted as communicative. An example of the latter is a passage from File 16, where the narrative frame of the role-play changes from dolls and a doll’s house, to imagined events within the kindergarten itself, where the children “act” themselves, and correspondingly change from UEN prosody to that of their native Tromsø dialect. (The main antagonist, a lurking thief, remains the same throughout both frames.)

### 4.2 Compounds—adult reference baseline data

The APs from the adult reference baseline data that satisfied the criteria in Section 3.3 were pitch normalized (centered around 0). The contours were smoothed and their timing were normalized so that the total duration and the syllables in every contour within the same group (e.g., “fire-/waterman (focal)”) were synchronized.

The mean of the CC across all possible pairs within each category in the adult baseline reference data was calculated as a test for internal consistency (i.e., to which extent the F0 contours correlate with each other). Averaging the internal consistency of all categories, gave a CC of 0.85, ranging from 0.67 for *vannmann* (non-focal) to 0.96 *brannmannen* (non-focal) (see columns 1 and 2 in Table 4).

**Table 4.** From the Reference Baseline Data: Internal CC Means for FIRE (col. 1) and WATER (col. 2) Compounds, CC Means of FIRE and Corresponding WATER Compounds (col. 3), and the Difference between col. 1—col. 3 (col. 4).

	CC FIRE (*brann*-)	CC WATER (*vann*-)	CC FIRE/WATER	Difference
-*bil* (focal)	0.91	0.85	0.66	0.24
-*bil*	0.82	0.87	0.29	0.54
-*bilen* (focal)	0.87	0.74	0.54	0.33
-*bilen*	0.83	0.82	0.35	0.48
-*mann* (focal)	0.95	0.82	0.69	0.25
-*mann*	0.77	0.67	0.31	0.46
-*mannen* (focal)	0.92	0.86	0.65	0.27
-*mannen*	0.96	0.85	0.54	0.41
-*mennene* (focal)	0.89	0.87	0.62	0.28
-*mennene*	0.81	0.87	0.32	0.49
-*stasjonen* (focal)	0.86	0.83	0.59	0.27

CC: correlation coefficient.

**Figure 5.**
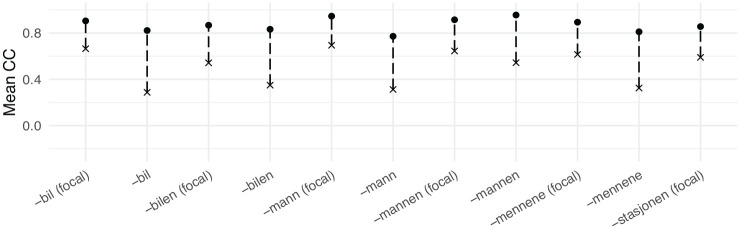
Difference in correlation coefficients for reference contours. Internal consistency CC for fire- compounds (dot), and averaged CC between fire- compounds for every water- compounds of the same category (x), and difference between the two (dashed line).

In the following section, the fire- compounds from the corpus data will be compared with the average adult baseline reference fire- and water- compounds, and the respective CCs will be calculated. To give an idea of what a UEN target situation may look like, the mean internal CC of each category of fire- compound has been compared with the mean CC between each fire- compound contour and every corresponding water- compound contour (e.g., the CC for each contour of “firetruck (focal)” and every “watertruck (focal)” has been calculated and averaged, column 3 in Table 4). These CCs are plotted alongside the internal CC of every fire- compound in Figure 5. Since they share the peak in the last syllable (compare the two contours in Figure 5), it is expected that the F0 contour of the fire- compounds is somewhat correlated with the water- compound contours (between 0.29 and 0.69, x’s in Figure 5, column 3 in Table 4). Bearing in mind that the children’s production is uncontrolled as opposed to the adult baseline reference data, one should still expect the contours to be more correlated with the corresponding baseline fire- compounds than the corresponding baseline water- compounds if the production is target-like. In summary, the correlation coefficient of children’s fire- compounds and the baseline fire- compound (henceforth “ fire-CC”) should be higher than the correlation coefficient of children’s fire- compounds and the baseline water- compounds (henceforth “ water-CC”). It follows from this that when the water-CC is subtracted from the fire- compound, the difference should be positive if the production is target-like (as in Table 4, column 4, for the adult baseline reference data, where the differences range from 0.49 to 0.24). If the children have generalized the c-dialect pattern to the RPR, the opposite should hold, and the difference should be negative.

**Figure 6.**
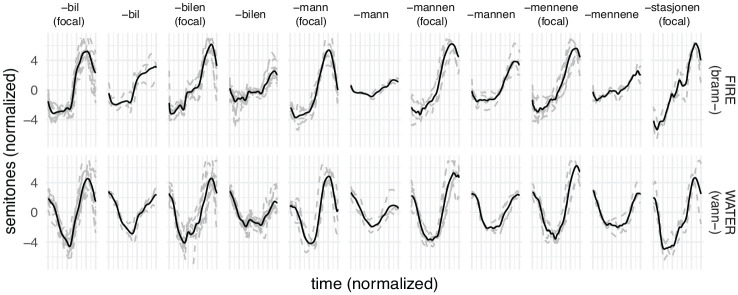
F0 contours of reference values, with mean (black solid) and individual (gray dashed) contours.

The actual F0 of the adult baseline reference contours, along with mean F0 contours for each category, are given in Figure 6.

### 4.3 Compounds—corpus data

A total of 90 fire- compound APs satisfied the criteria outlined in Section 3.3. The 90 F0 contours were compared with the corresponding fire- and water- compounds (means) in the baseline reference data, based on focal/non-focal and the number of syllables, and distribution of long and short vowels (*brannst*[i:]*gen*, “ladder,” and *brannsl*[a]*ngen*, “hose,” were compared with *brannb*[i:]*len* and *brannm*[a]*nnen*, respectively). An exception was APs with an additional unstressed word (for instance *brannbil nå*, “firetruck now”), where the length of the vowel of the additional word was not taken into account and counted as short regardless.

The value of the F0 contours rev was normalized (centered around 0). The contours were smoothed and their timing was normalized and synchronized using the same parameters as the corresponding fire- and water- baseline reference contours to make the calculation of the CC possible.

**Figure 7.**
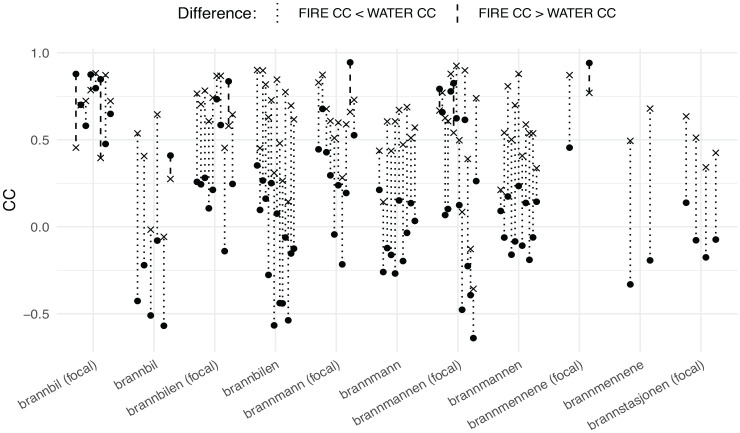
Correlation coefficients (CC) between individual F0 contours from the corpus and corresponding reference baseline contours. The cases where the fire-CC (dot) is higher than the water-CC (x) are indicated by dashed lines (more target like). The opposite cases are indicated by dotted lines (less target-like).

The functional CCs were calculated and are given in Figure 7, and tabulated for each child in Table 5 (median, interquartile range, and difference between medians). The F0 contours are given in Figure 7, along with the baseline reference contours (in bold for ease of exposition) and pointwise median and interquartile range for the corpus data in cases with four or more contours. The contours with higher water-CC than fire-CC have been plotted alongside the baseline reference water- compound contours, and vice versa.

**Table 5.** Correlation Coefficient Medians and Interquartile Range for Fire- (Target) and Water- (Non-Target) Compounds, with Differences (Positive = More Target-Like).

Subj	*n*	Median.*vann*	*iqr.vann*	median.*brann*	*iqr.brann*	Diff
Celice	26	0.632	0.305	0.089	0.381	−0.543
Hedda	4	0.775	0.233	0.405	0.472	−0.371
Inga	1	0.677	0.000	0.429	0.000	−0.249
Kimbo	4	0.541	0.093	0.085	0.316	−0.456
Klara	4	0.560	0.245	0.522	0.594	−0.039
Lars–Lars	38	0.541	0.299	0.024	0.662	−0.517
Morten	14	0.710	0.209	0.178	0.568	−0.531

**Figure 8.**
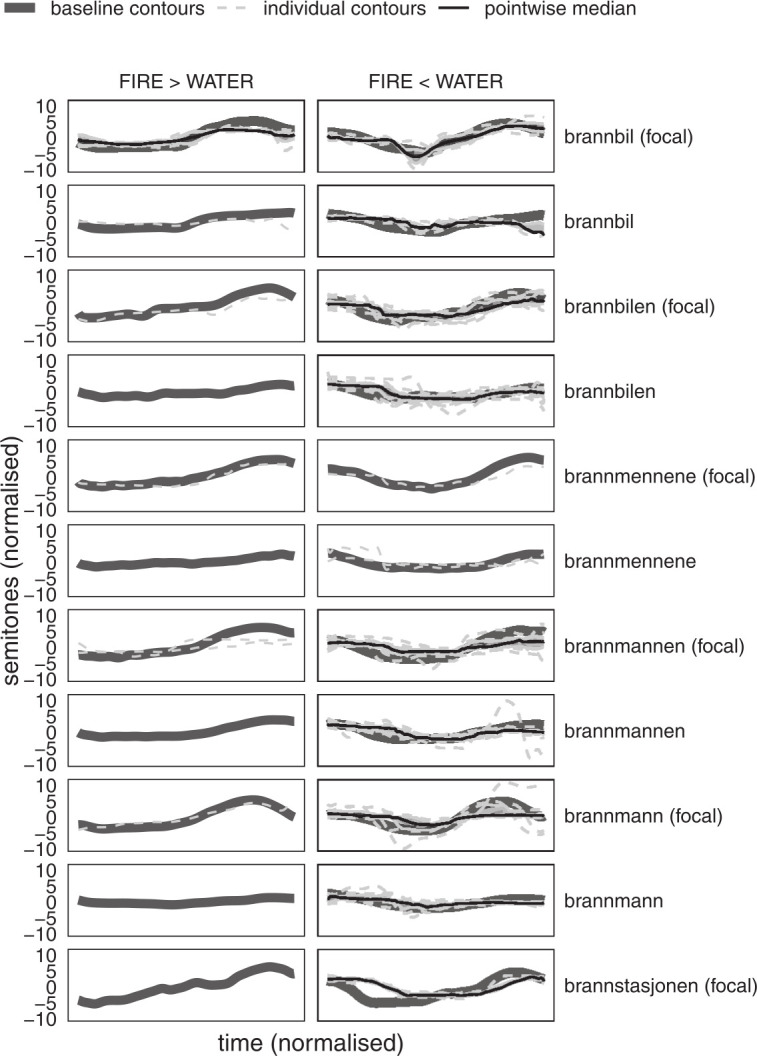
Individual F0 contours of fire- compounds from the corpus (light gray) compared with mean baseline contours (dark gray). Contours with higher fire-/water-CC are compared with baseline fire-/water- compounds, respectively (compare Figure 7). Pointwise median (black solid) and pointwise interquartile range (IQR, shaded area) are given where *n* contours > 3.

As shown in Figure 7 and Table 5, and visualized in Figure 8, the majority of the F0 contours from the corpus data have a higher water-CC than fire-CC and can therefore not be characterized as target-like UEN. Likewise, the production of all the children was, on median, not target-like, except for Klara, whose production can be characterized as chance level, and Inga, from whom only a single AP (which happened to be not target-like) satisfied the above criteria.

## 5 Discussion

The phonetic contours of the auto-divergent register (RPR) had clear features of UEN, phonetically. The extent to which they appear target-like varies, both sporadically and creatively, and is a subject for further investigations. What this investigation indicates with acoustic data is that the children in the study use phonetic/acoustic features from UEN prosody as a metacommunicative code to signal in-character role utterances. The reports mentioned in Section 1.2 above have thus been demonstrated to hold.

This was the foundation for the research question, which asked for the domain of tonal accents in compounds:

- RQ: To what extent is the morpho-phonological distribution in children’s RPR “target like” Urban East Norwegian?

Although the results are somewhat mixed, there is no indication that any of the children had command of the UEN (d-dialect) tonal accent differences in compounds. On the contrary, five of the six children who produced any data to speak of, seemed to overgeneralize from their native c-dialect’s compound accent (accent 2), whereas the last (Klara) performed at something that could at best be characterized as chance level. Note that the F0 contour of accent 2 in UEN and the Tromsø dialect are almost opposites (see Figure 1 above). It is very obvious that they have code-switched to a different dialect, phonetically, and that they use the “correct” F0 contour but on the “wrong” words.

Although more controlled investigations are warranted, we can put forward two possible explanations for the data: (a) North Norwegian children’s RPR has a compound accent (like the Tromsø dialect), and/or (b) differential tonal accent marking on compounds needs more positive input to be acquired, than that available to North Norwegian children.

To be clear, the first explanation does not exclude the possibility that children do have the differential tonal accent marking in compounds as a part of their linguistic competence, but that RPR, as a register, differs from UEN in this regard. Put differently, the target of children’s RPR may not be UEN for this part of grammar, or rather: the grammar of their RPR is *phonetically* but not *morpho-phonologically* like UEN. Given the auto-diverging aspect of RPR, this is not a very unreasonable assumption, as one could make the case that the phonetic features (the F0 contours) are more salient than the morpho-phonological distribution of those contours:^9^ first, the acoustic features are manifested in almost every prosodic foot, whereas the differences in morpho-phonological distribution are manifested only in a subset (e.g., in compounds). Second, the acoustic features could be seen in light of “the periodicity bias”: the bias that infants’ attention is drawn more toward voiced than unvoiced sounds (Cutler & Mehler, 1993), which has often been used to explain the correlation between prosodic awareness and reading measures (see, for instance, Wood & Terrell, 1998). For the meta-communicative code to be useful to the interlocutors, it should be easily picked up on, in which case more salient features with a wide distribution would rank over less salient ones. And third, the acoustic difference between the tonal accents of the Tromsø dialect and UEN may also make them easier to imitate than if they were more closely related, as demonstrated in studies of shadowing and imitation (Babel, 2012; Walker & Campbell-Kibler, 2015). These factors may also be arguments for the second, stronger explanation: less salient features, like accent marking in compounds, probably need more total positive input to be acquired, and the children’s input of UEN is, for most, atypical and sparse compared with that of their native variety. It may well be the case that the d-dialects’ differential accent marking of compounds remains unavailable to most speakers of a dialects with a compound accent throughout adulthood. It could also be a developmental phenomenon: Petrone et al. (2021) found a correlation between working memory and phonological prosodic differences in their study, thus cognitive development could also play a role, alongside input frequency.

(a) and (b) may also be two sides of the same coin: most of the input in RPR happens in role-play. If RPR is perceived or categorized *as* UEN by the children (consciously or not, i.e., RPR *is*, in the children’s ears, the variety they hear in television etc., and not something different), the input in RPR/UEN during role-play will be piled together with the input in *real* UEN from other sources, thus deteriorating the quality of the total positive input in UEN by diminishing the relative amount of compounds with differential accent marking.

The results of this study have potentially unveiled a limit to or bottleneck for the acquisition of tonal accent systems in a second dialect, even in young children, where the acoustic tonal contour of tonal accents or pitch accents are easier to acquire than their morpho-phonological distribution. Further research is warranted to investigate the degree to which this holds across situations, populations, and languages, in addition to the effect extra-linguistic factors, such as age and cognitive development. Furthermore, it would be interesting to scrutinize the extent to which such interfacing with morphology or syntax also plays a role for the acquisition of tone in a second dialect in canonical tone languages.

## 6 Conclusion and further research

This exploratory multiple case study has demonstrated that children from North Norway between the ages 3 and 4 have some command of the tonal accents of the Urban East Norwegian dialect (UEN), which they use in role utterances in role-play. This command, however, seems to be limited to the phonetic/acoustic properties of the tonal accent, and not so much the morpho-phonological properties, at least as far as tonal accents in compounds are concerned. This is evident from the non-target like overgeneralization of the compound accent (≈ accent 2) from the prosodic system of their own dialect (c-dialect), to the fire- compounds (tonal accent 1 in UEN, a d-dialect, where the tone accent in compounds vary) in their role utterances, as these were higher correlated with the UEN adult reference contours of tonal accent 2 (water- compounds) than tonal accent 1.

Two possible explanations for this were put forward and discussed: first, that the differential tone marking system of UEN, unlike the phonetic/acoustic properties of the variety’s prosody, is not a part of the North Norwegian RPR; second, that the differential tone marking system is unavailable to most Norwegian speakers of a c-dialect due to the low salience of the features, which is connected to the restricted distribution and thereby low frequency in the input.

As this study is explorative, its conclusions should be tested and attempted to be replicated in a more controlled (experimental) study. This could be done, for instance, by combining the methodology from Andersen’s (1990/2014) use of hand puppets to elicit sociolinguistic registers in spontaneous role-play utterances, with the more controlled environment of the shy puppet paradigm (e.g., Guasti et al., 1995): we can coin this as “the anthropophobic puppet” paradigm, where children themselves have to use a hand puppet to interrogate the experimenter’s hand puppet who is afraid of, and therefore does not want to talk to humans. This methodology has been piloted by the present author, and it has yielded mixed but encouraging results for eliciting RPR and should be tested further.

The results of the present paper also open up for several other questions. First, there is the question of the native(like-)ness of children’s use of UEN, which could be tested by playing excerpts of children’s role utterances to native raters. Of major importance here is the selection of excerpts in terms of controlling for (lexical, morphological, phonological, etc.) features that differ between the varieties, and making sure the selection is representative, which solicits a longer discussion and consideration.

Second, there is a question of development: the fire- compounds in this study were elicited from 3 to 4 years of age, approximately. Will they have acquired the differential accent marking later, and what kinds of and/or amounts of input are required for them to do so?

Third, only production has been gauged. A remaining question is whether or to what degree Northern Norwegian children, who do not make tonal accent distinctions in compounds, be able to exploit UEN tonal accent to distinguish minimal pairs such as 2 ballrom (“ball pit”) and 1 ballrom (“ball room”)?

Another tenet of this paper is the use of functional data analysis in empirical questions of Scandinavian tonal accents, which should be explored further, and with the more exploratory statistical procedures which are made possible within that framework of statistical analyses. This could yield new insights into the phonetics and phonology of the Scandinavian tonal accents.

## Appendix A

### Age of children at each recording session

**Table A1.** Recordings/Session Number and Ages (Years, Months, Days).

Ssn.no.	Ref.	Lars–Lars	Celice	Morten	Inga	Hedda	Kimbo	Klara
1	–	2; 11.16	2; 11.27		2; 11.20	3; 1.20	2; 10.23	2; 7.1
2	0.7	2; 11.23	3; 0.3		2; 11.27	3; 1.27	2; 10.30	2; 7.8
3	0.14	2; 11.30	3; 0.10	3; 4.7	3; 0.3	3; 2.3		
4	0.28	3; 0.13	3; 0.24	3; 4.21	3; 0.17		2; 11.20	2; 7.29
5	1.10	3; 0.26	3; 1.6	3; 5.6	3; 1.2	3; 3.2	3; 0.5	2; 8.11
6	1.20		3; 1.16	3; 5.16	3; 1.12	3; 3.12	3; 0.15	2; 8.21
7	2.6	3; 1.22	3; 2.2	3; 5.30	3; 1.26	3; 3.26		2; 9.7
8	2.13	3; 1.29	3; 2.9	3; 6.6	3; 2.2	3; 4.2	3; 1.5	2; 9.14
9	2.25	3; 2.10	3; 2.21	3; 6.18	3; 2.14	3;4.14		2; 9.26
11	3.17	3; 3.3	3; 3.13	3; 7.11	3; 3.7	3; 5.7		2; 10.18
12	3.29	3; 3.15	3; 3.25	3; 7.23	3; 3.19	3; 5.19		2; 11.0
14	4.27	3; 4.12	3; 4.23		3; 4.16	3; 6.16	3; 3.19	2; 11.28
15	5.3	3; 4.19		3; 8.27		3; 6.23		3; 0.4
16	6.22	3; 6.7	3; 6.18	3; 10.15		3; 8.11		3; 1.23
18	7.19		3; 7.15		3; 7.8		3; 6.11	3; 2.20
21	9.2	3; 8.18	3; 8.28		3; 8.22	3; 10.22	3; 7.25	3; 4.3
25	11.9	3; 10.1	3; 11.5			4; 0.29	3; 9.10	3; 6.10
27	1; 0.6	3; 11.22	4; 0.2	4; 3.30		4; 1.26		3; 7.7

Missing ages indicate absence from the recordings. The reference values indicate the time since the first recording.

## Appendix B

### Baseline reference data sentences

**Table B1.** Sentences for Baseline Reference Data Recording.

*Nei, det var en*	*BRANNMANN*,	*det jeg så i*	*brannbilen*,	*sa jeg!*
	*VANNMANN*		*vannbilen*	
	*BRANNBIL*		*brannstasjonen*	
	*VANNBIL*		*vannstasjonen*	
“no it was a	. . .	what I saw in	. . .,	I said”
*Nei, det var i en*	*BRANNBIL*	*jeg så en*	*brannmann, sa jeg!*	
	*VANNBIL*		*vannmann*	
“no it was in a	. . .	I saw a	. . .	I said!”
*Nei*,	*en brannbil*	*så jeg i*	*BRANNSTASJONEN*,	*sa jeg!*
	*en vannbil*		*VANNSTASJONEN*	
	*BRANNMENNENE*		*brannbilen*	
	*VANNMENNENE*		*vannbilen*	
	*brannmennene*		*BRANNBILEN*	
	*vannmennene*		*VANNBILEN*	
	*BRANNMANNEN*		*brannbilen*	
	*VANNMANNEN*		*vannbilen*	
	*brannmannen*		*BRANNBILEN*	
	*vannmannen*		*VANNBILEN*	
“no	. . .	I saw in	. . .,	I said!”

## Notes

1. Scholars disagree about the basis for the distribution of the accents across words, and this is not of importance to the present study (but see, for example, Kristoffersen, 2006, 2009; Lahiri et al., 2005; Wetterlin, 2010, for discussion).

2. Although the notion of salience merits further discussion, we will not indulge in that here.

3. Oslo, Stavanger, Bergen, Ålesund, and Trondheim (Fintoft, 1970).

4. One anonymous reviewer suggested investigating compounds starting with *poli’ti* “police” instead. The background for this suggestion is that compounds with roots like *poli’ti*, which have anacrusis/-es and final stress, *always* get accent 1 in compounds in UEN, as opposed to monosyllabic and trochaic words, where it varies. *poli’ti* may therefore be a more transparent exemplar and bigger generalization of the pattern to the children, and thus a more ideal testing ground for the hypothesis. However, apart from the fact that there are not by far as many compounds starting with *poli’ti* as there are compounds starting with *brann* in the corpus, the fire- compounds are an area in the grammar where the Tromsø dialect and UEN differ, and thus these structures represent a good way of exploring the (limits of) attainment of the UEN variety by children from Tromsø, even though it may pose a more difficult challenge than compounds with other words.

5. This is a possible source of error, as human alignment cannot perform with the same consistency, or at least unbiased automaticity, as, for instance, a forced alignment algorithm, especially in the cases where syllable boundaries are liquids or hiati. For geminates, a script was made in Praat to pinpoint the exact middle of a selection. This is in essence the same method as undertaken by Hognestad (2012).

6. In fact, it was not uncommon for the children to use their native dialect intonation instead of UEN in role utterances, or a combination of the two, starting in one and ending in the other. Others still were hard to categorize as one or the other at all, in particular in cases with agitated screaming. This intrasentential prosodic code-switching and, at times, ambiguity, makes it difficult to establish a meaningful estimate of the ratio of UEN prosody to Tromsø dialect prosody in the data.

7. The equation for the correlation coefficient from Li and Chow (2005) is as follows:

$$\rho (X,Y)=\frac{〈〈X-E(X),Y-E(Y)〉〉}{∥X-E(X)∥∥Y-E(Y)∥}$$

where $〈〈X,Y〉〉=\int_{a}^{b}X(t)Y(t)dt$ is the Euclidean inner product and $∥X∥=\sqrt{〈〈X,X〉〉}=\sqrt{\int_{a}^{b}x^{2}(t)dt}$ is the Euclidean norm (Li & Chow, 2005, p. 85, cf. Dubin & Müller, 2005, p. 874; Pini et al., 2019, p. 6; Ramsay & Dalzell, 1991, p. 546; Sangalli et al., 2009, p. 40). For the equation, a modified version of the function inprod.bspline from the fda package in R was used. It uses numerical integration by the trapezoidal rule with Richardson extrapolation as the approximation method; see the package documentation (Ramsey et al., 2020) and the R scripts in the replication data repository (Strand, 2021).

8. An anonymous reviewer pointed out that if the question was whether children’s RPR was morpho-phonological target-like UEN, then it would be more obvious to start by investigating the distribution of tonal accents in simplex words rather than in compounds. This is true, and the author welcomes any such attempts. The reason such an endeavor is not included in this paper is because it would be methodologically risky on spontaneous data: tonal accent in (simplex) word interact with many other factors, especially morphological, but probably also segmental, that may affect how felicitous children are in acquiring the tonal accent of any word (e.g., vowel length and presence of unvoiced stops). Finding and choosing words that would be comparable across both accents to test children’s attainment of both is not straightforward (not to mention the job of collecting adult UEN reference baseline data). The range of possible choices would have introduced increased researcher degrees of freedom, making the result less reliable (cf. Roettger, 2019).

9. The present author would argue that most Norwegian speakers, including speakers of North Norwegian varieties, are unaware of this phonological difference between c- and d-dialects.
